# Effect of Acoustic fMRI-Scanner Noise on the Human Resting State

**DOI:** 10.1007/s10548-022-00933-w

**Published:** 2022-12-19

**Authors:** Matthias Grieder, Thomas Koenig

**Affiliations:** Translational Research Center, University Hospital of Psychiatry and Psychotherapy, University of Bern, Bern, Switzerland

**Keywords:** EEG, Noise, Posture, Rest, Vigilance, Cortical

## Abstract

Our knowledge about the human resting state is predominantly based on either electroencephalographic (EEG) or functional magnetic resonance imaging (fMRI) methods. While EEG recordings can be performed in seated posture in quiet conditions, the fMRI environment presents a substantial contrast with supine and restricted posture in a narrow tube that is filled with acoustic scanner noise (ASN) at a chainsaw-like volume level. However, the influence of these diverging conditions on resting-state brain activation is neither well studied nor broadly discussed. In order to promote data as a source of sharper hypotheses for future studies, we investigated alterations in EEG-frequency-band power (delta, theta, alpha, beta, gamma) and spatial power distribution as well as cortical vigilance measures in different postures and ASN surroundings over the course of time. Participants (N = 18) underwent three consecutive resting-state EEG recordings with a fixed posture and ASN setting sequence; seated, supine, and supine with ASN (supnoise) using an MRI simulator. The results showed that compared to seated, supnoise, the last instance within the posture sequence, was characterized by lower power and altered spatial power distribution in all assessed frequency bands. This might also have been an effect of time alone. In delta, theta, alpha, and beta, the power of supnoise was also reduced compared to supine, as well as the corresponding distribution maps. The vigilance analysis revealed that in supine and supnoise, the highest and lowest vigilance stages were more dominant compared to the seated and earliest posture condition within the sequence. Hence, our results demonstrate that the differences in recording settings and progress of time are related to changes in cortical arousal and vigilance regulation, findings that should be taken into account more profoundly for hypothesis generation as well as analytic strategies in future resting-state studies.

## Introduction

The gradient coils of MRI scanners emit loud ASN during image acquisition. The peak volume intensity can range from 90 to 130 dB or more in the 3 T scanner types that are used in the clinic or for research (Price et al. 2001; Lee et al. 2017). The obvious discomfort caused by the noise can be mitigated by compulsory provision of earplugs to the person in the scanner, which lower the noise level by approximately 40 dB (Solana et al. 2016). However, fast gradient switching during echo-planar imaging (EPI), which is applied in most studies investigating changes in brain activity related to rest or any kind of perceptive or cognitive task, appears to cause the loudest noise. Thus, the noise remaining despite the use of earplugs still considerably impedes the optimal experimental conditions. Naturally, several attempts have been made to reduce ASN, including noise-cancellation audio systems, installation of better isolation, adjustment of gradient parameters, and introduction of new MRI concepts (Edelstein et al. 2002; Hennel et al. 1999; Cho et al. 1998; Wu et al. 2010; Weiger et al. 2013; Grodzki et al. 2012; Solana et al. 2016). However, this achieved only moderate success, as participants in the latest scanner models are still required to wear hearing protection (protecting only from airborne noise nonetheless).

Regardless, the question of whether mental states recorded in shielded MRI scanner rooms are comparable to mental states in everyday life or even in shielded EEG booths remains unresolved. In addition to ASN, the supine and strongly constricted posture of participants in a study in the scanner might also diminish the transferability of the results to all-day situations or experiments that are performed in a seated posture. As such, the effect of posture on task performance or resting state has been previously investigated. For example, Thibault et al. (2014) and Spironelli and Angrilli (2017) found that supine posture decreases beta (β) and gamma (γ) power of EEG frequencies compared to an upright seated posture. Such posture-dependent surface power alterations might be associated with changes in posterior cerebral spinal fluid volume (Rice et al. 2013). Nonetheless may these posture effects only partially generalize to the particular and heavily confined situation of subjects in an MRI scanner. However, the effects of ASN on brain activity are not well understood. While behavioral data can easily be compared inside and outside the scanner, neurophysiological data comparisons are more challenging. For example, data regarding fMRI resting-state brain activation can only be obtained inside the scanner, and only with ASN. A possibility to measure the influence of ASN on fMRI resting-state networks is to test acquisition schemes with different ASN levels (i.e., “silent” vs. “noisy”). Using this approach, Langers and van Dijk (2011) detected only marginal effects of ASN on resting-state dynamics. Unexpectedly, their “silent” acquisition scheme caused a more disturbed resting state (higher alertness and arousal) than the “noisy” (yet continuous) scheme. Hence, the authors concluded that varying noise levels and characteristics do not eliminate the confounding effect of ASN in resting-state fMRI data. A similar approach to acoustic stimulation was investigated by Yakunina et al. (2015), who observed ASN effects not only in auditory brain regions, but also in networks involving attention. In line with Langers and van Dijk (2011), Yakunina et al. (2015) suggested ASN as a nuisance factor in resting-state fMRI data.

Unlike fMRI, with EEG, it is possible to record brain activity inside and outside the scanner. Conversely, inside the scanner, there is a constant magnetic field regardless of whether an acoustically noisy sequence is running. Moreover, owing to the scanner’s need for permanent cooling, the helium pump emits acoustic noise, which does not allow complete silence as an experimental condition inside the scanner. Taken together, it is impossible to separate the factors of posture, ASN, and magnetic field using a real MRI scanner for this purpose. Another aspect is the definition of the human resting state. Before the discovery of fMRI resting-state networks, a common standard for measuring cerebral resting-state activity was a setting comprising a silent, dark, and shielded room, the typical EEG-lab setting. Considering ASN (amongst other environmental differences), it can be problematic to compare the resting state of an fMRI setting with that of an EEG setting.

In the current study, we used an MRI simulator to investigate the effect of posture and that part of the ASN that is perceived through the ears, without an actual magnetic field complicating the situation. The MRI simulator can be described as a scanner-shell with the necessary equipment to simulate an MRI measurement without actually recording data. This structure comprises a tube the same size as a real MRI, a stretcher with an electric motor to move it in and out, inside light and cooling, and the choice of several MRI-sequence noises from various manufacturers. Most importantly, recording an EEG is also possible without the need to account for magnetic field distortions. On the downside, no magnetic field implies that the well-documented effect of magnetic vestibular stimulation (MVS) on resting-state activity, induced in real MRI scanners, could not be accounted for in this study (Roberts et al. 2011; Boegle et al. 2020; Andreou et al. 2017).

For the purpose of this study, we compared the resting-state EEG of healthy participants under the experimental conditions of three postures: seated outside the MRI simulator, supine inside the MRI simulator, and supine inside the MRI simulator with a standard Siemens EPI-sequence noise overlapping with a helium pump noise (for better clarity and brevity, the conditions are hereafter termed as seated, supine, and supnoise). Note that this study does not allow disentangling posture and time (i.e. sequence) effects, since the sequence of the postures was fixed. Instead, effects observed in this study merely reflect resting state alterations of participants who are initially seated, then supine, and finally supine with the addition of ASN. We compared the EEG frequency spectrum and vigilance over time between these conditions.

Due to the lack of comparable studies, the current analysis of EEG data as a function of posture and ASN is exploratory. However, in line with Thibault et al. (2014), we expected decreased β and γ power in the supine posture compared to the seated posture. For the vigilance analysis, we anticipated lower vigilance stages to be more present than in the supine posture than in the seated posture. Of note, no hypotheses were postulated for the supnoise condition because of the available data being inconclusive.

## Methods

### Participants and Procedure

Eighteen healthy participants (eight females) were included in this study (mean age = 23.5 years, SD = 2.7), after one participant with restless legs syndrome was excluded (Akpinar et al. 2007). The inclusion criteria were as follows: age 18–45 years, willingness to participate, willing to provide written informed consent, right-handedness, German (or Swiss German) language as mother tongue, normal or corrected to normal vision, and luteal phase for women. The exclusion criteria were: past or current diagnosis of a psychiatric disease, any medical condition that could influence the brain’s physiology, intake of medication affecting neuronal activity, current or past drug abuse within the 2 years preceding the study, pregnancy, breast feeding, heart or brain surgery, any kind of metal objects in the body, claustrophobia, and Epworth Sleepiness Scale (ESS) > 10. This study was approved by the ethics committee of the Swiss canton Bern (KEK-Nr. 083/14), and was performed in accordance with the Declaration of Helsinki.

After the participants arrived at the study site, they completed the ESS questionnaire, and all women performed a pregnancy test. Next, the EEG was set up, and after completion, the resting-state EEG in the seated condition was recorded. The participants then lay down into the MRI simulator (Psychology Software Tools Inc., Pittsburgh, PA, USA), and a Siemens Style Mock Head Coil was attached. Following this, the resting-state EEG was measured in the supine posture in the MRI simulator, without any noise. The recording session was concluded by an additional resting-state EEG acquisition with scanner noise engaged using SimFx™ Software (Psychology Software Tools Inc., Pittsburgh, PA, USA). The ASN was emitted using the built-in speakers located in the base frame of the MRI simulator at a fixed volume level of 90 dB. We measured this particular volume level in advance at a real Siemens 3 T Prisma Scanner from the entrance to the scanner room (no MRI-safe volume-meter was available). Accordingly, the volume level set at the MRI simulator was measured from a comparable distance to the simulator’s tube. The resting-state EEG recordings in the seated and supine postures lasted for 6 min and 40 s each, and were divided into three 2-min eyes-closed periods, interrupted by two 20-s eyes-open periods. In the supnoise condition, no eyes-open periods were introduced. The participants were not aware of the purpose of this study (i.e., the comparison of three resting-state EEG recordings with different conditions), as the data for the current study were recorded preceding the data acquisition of the study Ruch et al. (2021), which involved several EEG recordings and lying in the MRI simulator. This circumstance led to the study design limitation, namely a fixed order of the posture and ASN setting as described above, without counterbalance.

### EEG Recording

The EEG recording setup was the same as in Ruch et al. (2021), with 22 sintered chloride ring electrodes mounted on an elastic cap and complying with the international 10–20 system (Fp1, Fp2, F3, F4, C3, C4, P3, P4, O1, O2, F7, F8, FT9, FT10, T5, T6, Fz, Cz, Pz, Oz, CP5, CP6). Cz was the recording reference and the ground electrode was located between Pz and Oz. These electrodes were connected to a 16-bit BrainAmp Standard amplifier (Brain Products GmbH, Gilching, Germany). An online band-pass filter (0.1–1000 Hz) was applied, at a sampling rate of 5 kHz. The amplifier’s input range was 3.3 mV. The impedances were kept below 10 kΩ, and the EEG was down-sampled offline to 1 kHz. Six additional adhesive electrodes were connected to a bipolar ExG BrainAmp 16 (Brain Products GmbH, Gilching, Germany) and attached accordingly for electrooculogram (EOGL, EOGR), electromyogram (EMGL, EMGR), and electrocardiogram (ECGL, ECGR). For this study’s purpose, only data from the 22 EEG channels were used.

### EEG-Vigilance Preprocessing

Preprocessing steps were performed in accordance with those recommended in the VIGALL manual (Olbrich et al. 2012b). In particular, the data were bandpass-filtered at 0.5–70 Hz, with an additional Notch-filter at 50 Hz. Eye movements and other re-occurring artifacts were removed using ICA. Next, data were recalculated to the average reference, and a first rough artifact screening was performed. Subsequently, the EEG was segmented into 1-s epochs, and the epochs containing artifacts were removed. Because of the requirement for a certain channel montage by the VIGALL algorithm, six channels were computed by topographical interpolation (splines = 4, degree = 10, lambda = 1E-05), and two were discarded as they did not match the guideline. The data were then further down-sampled to 512 Hz. Finally, the VIGALL add-in in the Vision Analyzer (2.1, Brain Products GmbH, Gilching, Germany) was run.

The VIGALL method (Olbrich et al. 2012b) was performed in accordance with previous studies. Vigilance stages are specified in a number of studies (e.g. Corsi-Cabrera et al. 2000; Tsuno et al. 2002). In brief, this method comprises seven stages; 0, A1, A2, A3, B1, B2/3, and C. Stage 0 are EEG epochs with low voltage without slow horizontal eye movements and reflects high alertness. Stages A1–A3 are characterized by alpha (α) oscillations in the frontal and central electrodes and represent relaxed wakefulness. Stage B1 shows a low-amplitude EEG without apparent α but horizontal eye movements, while stage B2/3 is characterized by increased delta (δ) and theta (θ) power. Either B-stages mirror increased drowsiness. Finally, stage C includes sleep spindles and K complexes, which are typical sleep markers. Olbrich et al. (2012a) provided a more detailed description of the VIGALL method, which was validated using fMRI and PET (Olbrich et al. 2009; Guenther et al. 2011).

### EEG-Vigilance Parameterization and Statistics

Two parameters were calculated from the output of the VIGALL algorithm:

1. Ratio of EEG-vigilance stages: The relative amount of stages A1, A2, A3, B1, and B2/3 over all artifact-free 1-s segments was calculated (number of segments of one stage/total number of segments).
2. Average EEG-vigilance level (Huang et al. 2015): In order to extract a value for the average vigilance level of the entire resting-state EEG, the vigilance stages were rescaled into a variable ranging from 1 (B2/3, lowest stage) to 5 (A1, highest stage).

The main posture effects of all the above-described parameters were investigated using the non-parametric Friedman test. Post-hoc tests were performed using the Wilcoxon signed-rank test, which tests whether the central tendency of two dependent samples are different. P-values that did not survive a multiple comparisons correction by Holm are also provided (Holm 1979). All statistical analyses were performed using SPSS (version 28, IBM Corp., Armonk, NY, USA).

### EEG-Frequency Preprocessing

The resting-state EEG raw data were processed using Vision Analyzer. First, the resting-state EEG raw data were band-pass filtered at 0.5–20 Hz, with an additional Notch-filter at 50 Hz. This filter was only used for the conduction of an independent component analysis (ICA), which allowed the elimination of eye movement and other recurrent artifacts. The weights obtained through the ICA were then multiplied with the data to minimize the contaminated data fed to the fast Fourier transformation (FFT). This data was not filtered further than the above-mentioned online band-pass filter of 0.1–1000 Hz.

Before this, several other preprocessing steps were performed. In particular, the average reference was computed, bad channels were topographically interpolated (splines = 4, degree = 10, lambda = 1E-05), remaining artifacts were marked by visual inspection, and the EEG was segmented into 2-s epochs. Only the eyes-closed epochs were used for the analysis. Subsequently, FFT was performed on the voltage using a Hanning window of 10%. After zero-padding the epochs to length 2048 ms, the resolution was 0.488 Hz. Finally, each individual’s data was averaged over all segments and frequency points with these frequency bands: delta (δ) 0.5–4.0 Hz; theta (θ) 4.0–8.0 Hz; alpha (α) 8.0–12.5 Hz; beta (β) 12.5–30.0 Hz; gamma (γ) 30.0–48.0 Hz.

### Statistics of Frequency-Wise Topographic and Power Differences of Posture

To investigate the influence of posture and noise on the neuronal generator configuration at rest, we conducted five topographical analyses of variance (TANOVA) for the frequency bands δ, θ, α, β, and γ. TANOVA is a non-parametric randomization test that compares time-point by time-point topographical maps, or, more specifically in this study, frequency-band-wise power maps between the three posture conditions, seated, supine, and supnoise. Five thousand randomizations were run per TANOVA using Ragu software (Koenig et al. 2011). Post-hoc t-tests were conducted within the frequency bands to identify the significant main effects of posture condition. In addition, t-tests were used to investigate the power differences between postures in each frequency band. For this purpose, the root-mean-square (RMS) of all EEG channels per frequency band and posture was computed. Changes of t-test significance owing to correction for multiple testing are reported as well.

## Results

### Effect of Posture and Noise on EEG-Vigilance Stages

**Fig. 1:**
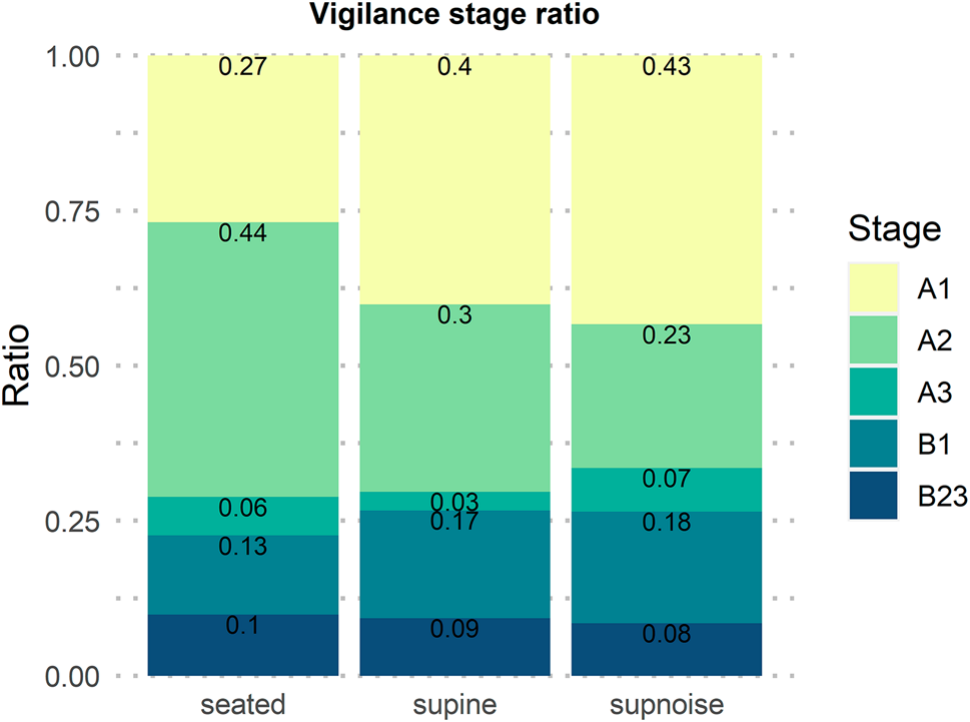
Mean ratios of EEG-vigilance stages of the postures seated, supine, and supnoise

The Friedman test comparing all three postures showed significant differences in stages A1 (*Χ*^*2*^ = 10.2; *df* = 2; *p* = 0.0060; power[1-β err prob] = 0.8244), A2 (*Χ*^*2*^ = 8.6; *df* = 2; *p* = 0.0132; power[1-β err prob] = 0.7505), and B1 (*Χ*^*2*^ = 6.2; *df* = 2; *p* = 0.0450; power[1-β err prob] = 0.5991). Post-hoc Wilcoxon test results yielded significant ratio differences between seated and supine (*Z* = − 3.0; *p* = 0.0012) and seated and supnoise (*Z* = − 2.9; *p* = 0.0023) in stage A1. Stage A2 showed an analog pattern with ratio differences between seated and supine (*Z* = 2.2; *p* = 0.0304), and seated and supnoise (*Z* = 3.3; *p* = 0.0003). Stage B1 differed only between the seated and supnoise (*Z* = − 2.2; *p* = 0.0261). Correcting the post-hoc tests for multiple comparisons had the effect that stage A2 difference between seated and supine was a tendency to significance only (*p* = 0.0522). The same applies to stage B1 effect between seated and supine (*p* = 0.0522). The other effects remained significant. Figure 1 illustrates these ratio differences in vigilance stages between the three different posture conditions.

### Frequency-Wise Topographic and Power Differences of Posture

**Fig. 2:**
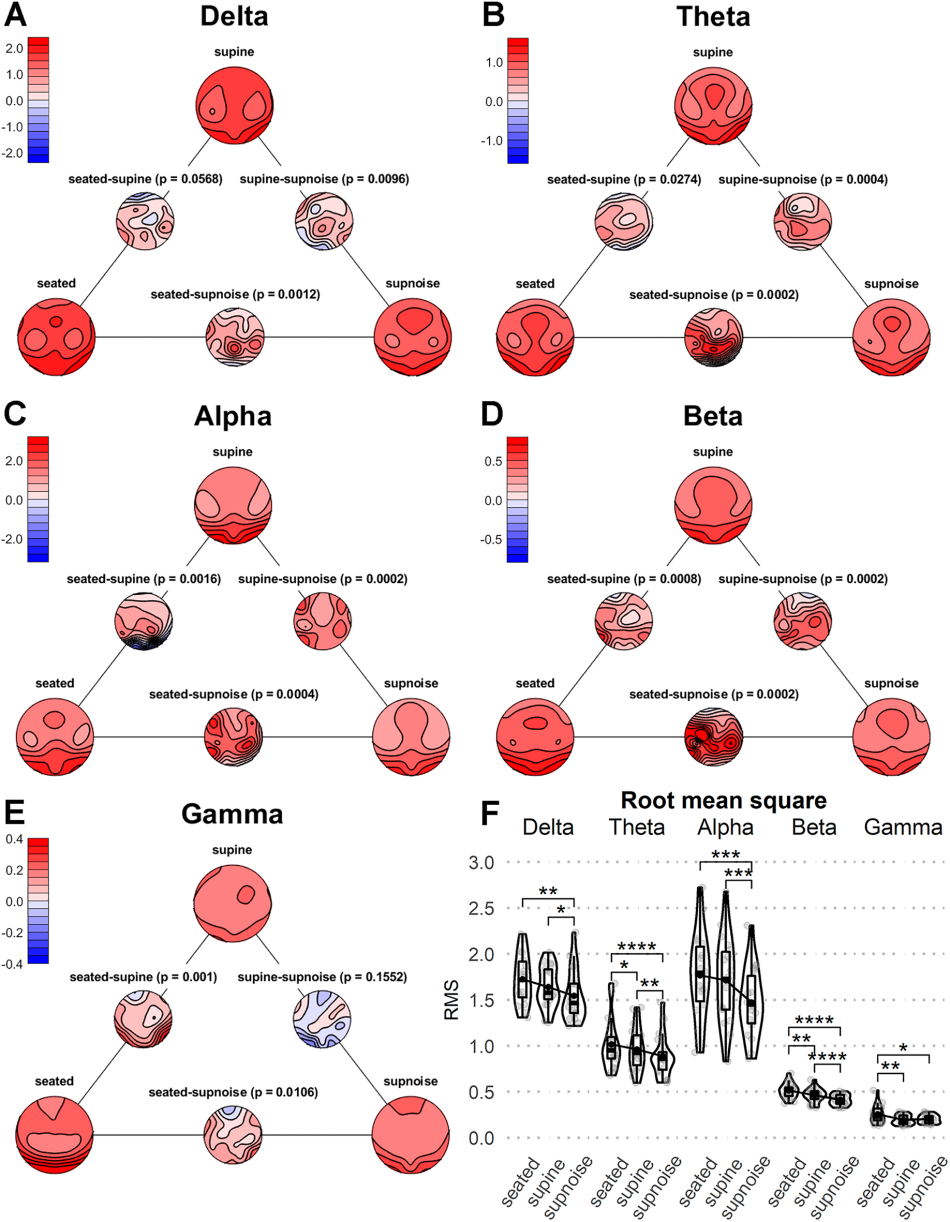
**A**–**E** illustrates power maps of each analyzed frequency band for the experimental conditions seated, supine, and supnoise. Between the power-maps, topographical t-maps (in color-steps of 1 t) of the statistical difference are depicted, including p-values. All of the significant p-values survive a Holm’s correction for multiple testing with each frequency band. **F** shows individual data points of mean RMS (grey dots), superimposed by corresponding line plot (sample mean), boxplot (interquartile range from 25 to 75%; black horizontal line represents median; whiskers indicate smallest and largest values within 1.5 times interquartile range below or above 75th percentile) and violin plot (density distribution). Horizontal bars on top of the plots indicate significant mean RMS differences tested with Wilcoxon signed-rank test (*p < 0.05; **p < 0.01; ***p < 0.001; ****p < 0.0001). Effects labeled with * (*p* < 0.05) did not remain significant after correcting for multiple testing

All five TANOVAs indicated a significant main effect of posture (*p* = 0.0004, Bonferroni corrected *p* = 0.0020). Figure 2A–E illustrates the post-hoc t-test results including topographical t-maps. In δ, it can be observed that the main posture effect was explained by altered topography in the supnoise compared to the seated or supine posture. The topographic distribution showed decreased power over the central parietal electrode and higher occipital power in the supnoise posture. The θ posture effect was characterized by an even more pronounced occipital power increment and centro-parietal decrease in the supnoise posture compared to seated, although all posture topographical maps differed significantly. In, the topographical difference between seated and supnoise appeared comparable, whereas a pronounced increase in occipital power from seated to supine was observed. Nevertheless, the topography between supine and supnoise differed with diminished lateral frontal and posterior power in supnoise compared to the supine posture. The β t-maps showed a pattern similar to θ in all three difference maps. Finally, γ showed differences only in the seated posture compared to supine and supnoise, whereas the supine and supnoise postures did not yield any significant difference. The main characteristic was a lower power over the frontal electrodes in the seated posture than in either supine condition.

When inspecting the RMS comparison, all frequencies except γ yielded lower values in the supnoise compared to the seated and supine postures. A decreased RMS in the supine posture compared to the seated posture was observed in θ, β, and γ. Exclusively in γ, ASN did not seem to be related to any power differences compared to supine. The differences between supine and supnoise in δ (*p* = 0.0920), between seated and supine in θ (*p* = 0.0920), and between seated and supnoise in γ (*p* = 0.0744) did not remain significant after correction for multiple testing.

## Discussion

The findings of the current study have a series of relevant implications for the interpretation of resting-state brain activity observed in an active MRI scanner. They indicate that the scanner environment along with the progress of time systematically affected organization of resting-state EEG activity and associated vigilance stages in our group of healthy young subjects. This organization and regulation of spontaneous brain-activity is on one side known to be altered by various important factors such as age (Koenig et al. 2002) and gender (Tomescu et al. 2018), and neuropsychiatric conditions such as the presence of ADHD (Strauß et al. 2018), depression (Hegerl et al. 2012), mania (Wittekind et al. 2016) or dementia (Smailovic et al. 2019). On the other side, nearly all brain information processing is affected by the brain’s background functional state (McCormick et al. 2015; Huang et al. 2017). In an MRI environment, changes in information processing that are associated with such factors as age, gender, or neuropsychiatric conditions must thus be considered as the product of an interaction between the task-demand itself, and the way this task-demand interacts with how particular groups of subjects adapt to the MRI environment.

In our analyses, we assessed the EEG power of common frequency bands. In addition, we explored topographical modulations within the frequency bands stratified by the three posture conditions. Next, we assessed differences in vigilance stages between the seated, supine, and supnoise postures, which provide a reliable measure of dynamic brain arousal changes (Huang et al. 2015). Hence, we will first discuss the power findings, followed by implications raised by the vigilance results.

The frequency-band power analysis confirmed the previously circumscribed and anticipated decrease in β and γ power in the supine compared to the seated posture (Thibault et al. 2014; Spironelli and Angrilli 2017). In addition, our data showed decreased global power in the θ band (Fig. 2F). While this runs against findings of increases in δ and θ power in the supine posture found e.g. by Spironelli et al. (2016), this difference may be explained by the fact that our subjects were not resting in a bed but in the rather unusual environment of an MRI simulator. Moreover, other than in most comparable studies, the supine posture always followed and never preceded the seated posture. Sequence effects might therefore explain some of the findings. Thus considering our particular study design, the finding of decreased θ power in supine posture, indicating a higher vigilance or lower drowsiness, appears unexpected. However, given the clear limitation of our design, such a conclusion would need an independent replication with a design with randomized sequences of posture conditions. Comparing power topographies (Fig. 2A–E), we also found occipital β and γ reduction in the supine posture compared to the seated posture, as reported by Thibault and colleagues (2014). However, we found an increase in frontal electrodes instead of a decrease in the two frequency bands, which contradicts the findings of Thibault et al. (2014). These local increases can be identified on the maps in Fig. 2D, E labelled “seated-supine,” showing blue (i.e., negative) t-values over frontal electrodes. Since the frontal increase in power in supine compared to seated can be seen in all the frequency bands we analyzed (δ, θ, and α were not analyzed in Thibault et al. 2014), this might be a finding that could be related to the particular recording setting in the MR simulator in the combination with elapsed time. Since this is a highly speculative interpretation, such a finding needs replication.

Our exploratory power analysis indicated that the introduction of ASN to the supine posture, as well as sequence effects of time that might have generated a general confound, revealed complex results. Conversely, global power was lowest in supnoise in all frequency bands except γ (Fig. 2F). The global power difference in supnoise was most likely explained by decreased power, mainly over central parietal electrodes, as illustrated by t-maps in Fig. 2A–D. Conversely, the same t-maps indicate occipital increases in power in δ and θ, to mention the most distinct. This finding might be related to the higher ratio of drowsiness in supnoise, as a consequence of the fixed recording setting order with supnoise in the last position. Furthermore, as outlined in the introduction of the EEG vigilance stages, δ and θ are more pronounced in a state of drowsiness, B2/3, despite the finding that total δ and θ power were lowest in supnoise.

Thus, not only the supine posture but also supnoise seem to interact with the power of the resting brain activity, in one or the other direction depending on the brain region. To make the image even more intertwined, frequency bands appear to be distinctly affected by the influence of posture, noise, and elapsed time. For example, δ and α power were not affected by posture and time, yet adding ASN with more elapsed time elicited power changes. In contrast, γ power did not change with the introduction of ASN, but was altered by the supine posture. In θ and β, both posture and noise possibly with the interaction of the confounded sequence effect influenced the power. To the same extent as addressed above, frequency-specific topographical changes might as well have been generated by time alone, a possibility that this study cannot rule out.

Our hypothesis that vigilance would be lower in the supine than in the seated posture was confirmed by the fact that drowsiness (B1 and B2/3) was more dominant in the supine postures. This effect would be also expected from elapsed time alone. In our view, this is one of the main implications of this study that follows from the observation that our study participants spent, on average, more than a quarter of the time in states classified as either B1 or B2/3 (Fig. 1), which are biological indices of drowsiness and transition to sleep onset (Sander et al. 2015). Behaviorally, these states are characterized by lowered attention and alertness, and correlate with a loss of control over logic of thought (Yang et al. 2010). The structure, the “mode of operation” and the mental correlates of self-organizing functional resting-state brain networks recorded in these circumstances may thus be systematically different from the states that determine our typical everyday behavior. The sometimes sought connection between variance and abnormalities in fMRI based resting-state connectivity and variance and abnormalities in individual behavior outside of the scanner (e.g., Menon 2011; Woodward and Cascio 2015; Bolton et al. 2020) may thus be mediated by brain mechanisms that regulate vigilance, and individual variance or abnormalities in these mechanisms. The fact that this particular finding might have been related to time alone raises the need for replication in future studies with counterbalanced posture and ASN settings. However, neither ASN nor time had any influence on the B1 or B2/3 ratio (Fig. 1).

In light of a possible sequence effect confound in our data, we should not ignore that some findings did not concur with what the literature would predict (Hegerl et al. 2012; Strauß et al. 2018) from a vigilance time course alone, whereas others did. Despite the fact that our design makes this unexpected observation inconclusive, it is still informative to compare our time-course of EEG changes to what the literature would predict, which might yield hypotheses for future studies about the direction of the effects. We found that stage A1 was more dominant in the supine or supnoise postures compared to seated. This indicates that the conditions in the MRI simulator were accompanied by a high alertness ratio, opposing a mere time-based hypothesis of a gradual decay of alertness with time (Olbrich 2012b). In contrast, stage A2 showed the highest ratio in seated with decreasing ratios in supine and supnoise. Yet, the lower ratio was only significant in supnoise compared with seated. In the normally expected gradual decrease of alertness in a normal environment, one would expect that A2 stages first increase at the “cost” of A1 stages, and may later be replaced by more stages of type B1 or B2/3. However, in our data, the relatively large decrease of A2 in later conditions might be explained only to a small part by an increase of stage B1, whereas the increase of stage A1 seems to account for most of this decrease. Hence, an increased A1 at the cost of A2 ratio paired with a higher B1 ratio in supine and supnoise compared to seated might reflect both a more alert rest and more pronounced drowsiness. However, which effects were driven by the interaction of time and/or posture could not be disentangled with the available data of this study. The implication that still can be taken is since stages A1 and A2 can be described as most closely related to “an awake state,” those would be the relevant stages to be included in analyses focusing on awake resting states.

This study has limitations that are addressed here. One might argue that ASN should be distinguished between ASN effects on structure-borne and airborne noise and that our data only accounts for airborne noise. Moreover, as outlined in the introduction, effects of MVS on resting-state vigilance or frequency power could not be investigated without an actual magnetic field. The main limitation of our study is however that some of the effects may be confounded or explained alone by the fixed sequence of the experimental conditions. As discussed above, some of the effects observed ran against the expectations of EEG changes associated with a time course of awake resting state. With time, one would typically expect a transition to lower levels of vigilance, but our data showed no general decrease of the mean vigilance level across the sequence of the three experimental conditions. Instead, we found that namely in the supine posture with ASN, there were both more states associated with higher (A1) and with lower (B1) levels. A similar point can be made for the frequency domain data, where i.e. the decreases in δ and θ are against what a mere progression of time in in a resting state would predict. This makes us think that the mere sequence of the conditions does not yield a reasonable explanation of our effect*s*, while the type of condition does.

With these vigilance findings, the above outlined implications gain further relevance by the observation that in our data, vigilance regulation was systematically altered depending on the experimental conditions and time. In other words, our data confirms, rather unsurprisingly, that we cannot simply observe resting-state brain activity per se, but must instead measure the way an individual’s spontaneous brain activity adapts to a given situation, which is further subject to changes with progress of time. Importantly, however, the environment that is constituted by an MRI scanner seems to present a situation in which the participant adapts in a way that is systematically different from situations that are ecologically more valid for most of the typically studied conditions. This issue should be taken into account when interpreting resting-state data and might be especially relevant in populations with systematically affected vigilance regulation (e.g., depression, attention-deficit/hyperactivity syndrome). In conclusion, we encourage the performance of future research to reinforce the much underrepresented discussion of drowsiness in resting-state fMRI studies (https://www.webofscience.com/wos/woscc/summary/367095b6-7a66-4aff-bfe9-e6eb9d266cfd-02547779/times-cited-descending/1).

## Data Availability

The datasets analyzed during the current study are available from the corresponding author on reasonable request.
